# Total Antioxidant Capacity and Total Oxidant Status in Saliva of Periodontitis Patients in Relation to Bacterial Load

**DOI:** 10.3389/fcimb.2015.00097

**Published:** 2016-01-06

**Authors:** Taowen Zhang, Oleh Andrukhov, Hady Haririan, Michael Müller-Kern, Shutai Liu, Zhonghao Liu, Xiaohui Rausch-Fan

**Affiliations:** ^1^Department of Science and Education, Yantai Stomatological Hospital, Binzhou Medical UniversityYantai, China; ^2^Division of Conservative Dentistry and Periodontology, University Clinic of Dentistry, Medical University of ViennaVienna, Austria

**Keywords:** oxidative stress, periodontopathic bacteria, saliva, total oxidant status, total antioxidant capacity

## Abstract

The detection of salivary biomarkers has a potential application in early diagnosis and monitoring of periodontal inflammation. However, searching sensitive salivary biomarkers for periodontitis is still ongoing. Oxidative stress is supposed to play an important role in periodontitis progression and tissue destruction. In this cross-sectional study, we investigated total antioxidant capacity (TAC) and total oxidant status (TOS) in saliva of periodontitis patients compared to healthy controls and their relationship with periodontopathic bacteria and periodontal disease severity. Unstimulated saliva was collected from 45 patients with generalized severe periodontitis and 37 healthy individuals and the TAC/TOS were measured. In addition, salivary levels of *Aggregatibacter actinomycetemcomitans, Porphyromonas gingivalis, Tannerella forsythia, Treponema denticola*, and *Fusobacterium nucleatum* in saliva were measured. Salivary TAC was lower in periodontitis patients compared to healthy controls. Moreover, a significant negative correlation of salivary TAC with clinical attachment loss was observed in periodontitis patients. No significant difference in the salivary TOS was observed between periodontitis patients and healthy controls. Bacterial load was enhanced in periodontitis patients and exhibited correlation with periodontal disease severity but not with salivary TAC/TOS. Our data suggest that changes in antioxidant capacity in periodontitis patients are not associated with increased bacterial load and are probably due to a dysregulated immune response.

## Introduction

Periodontitis is an inflammatory disease caused by specific bacteria and is characterized by gingival bleeding, periodontal pocket formation, destruction of connective tissue attachment, and alveolar bone resorption (Armitage, 1995). Periodontitis is initiated by the sub-gingival biofilm, but the progression of this destructive disease appears to depend upon an abnormal host response to those organisms (Cekici et al., 2014; Hasturk and Kantarci, 2015; Meyle and Chapple, 2015). Some specific bacteria such as *Aggregatibacter actinomycetemcomitans, Porphyromonas gingivalis, Tannerella forsythia, Treponema denticola*, and *Fusobacterium nucleatum* are strongly related to periodontitis (Socransky et al., 1998; Signat et al., 2011).

The classical parameters for diagnosis of periodontitis are clinical parameters such as probing depths of the gingival crevice, bleeding on probing, clinical attachment levels, and radiographic analysis (Goodson, 1986). These parameters are reliable to assess the severity of periodontitis under the condition of significant tissue destruction. However, measuring these parameters has poor prognostic impact (Lindhe et al., 1983) and therefore oral fluids have been investigated as an alternative diagnostic and prognostic approach (Kinney et al., 2011). In particular, salivary analysis has the potential to reflect current disease activity and severity, which can be advantageous for providing information used in risk assessment and monitoring of disease progress (Miller et al., 2010; Kinney et al., 2011).

Recently the advanced researches in molecular mechanisms in the pathogenesis of periodontitis have provided information about the specific biological pathways and biomolecules that could be used as biomarkers for risk assessment, diagnosis, and prognosis (Kinney et al., 2011). Obviously, most studies focused on markers that hold potential diagnostic significance relevant to three important biological phases of periodontal disease: inflammatory phase, connective-tissue degradation phase, and bone-turnover phase (Miller et al., 2010). Moreover, many factors associated with host-immune reaction to periodontal pathogens have been detected in saliva of periodontitis patients, for instance cytokines, chemokines, enzymes and immunoglobulins (Lamster and Grbic, 1995; Kaufman and Lamster, 2000; Seymour and Gemmell, 2001; Lamster et al., 2003). Nevertheless, studies about salivary biomarkers for the assessment of periodontitis are still ongoing (Giannobile et al., 2009).

Oxidative stress is defined as an imbalance between the production of reactive oxygen species (ROS) and the antioxidant capacity of organism. As most inflammatory diseases, periodontitis is characterized by oxidative stress, which might contribute to the host tissue destruction (for review, see Chapple and Matthews, 2007). Salivary markers of oxidative stress are extensively discussed as a possible tool for periodontal diagnostic (Tóthová et al., 2015). Since most oxidants have a very short half-life time, the measuring of oxidation products is widely used as an indicator of oxidative stress (Palmieri and Sblendorio, 2007). Previous studies show that periodontitis patients have the increased salivary levels of lipid peroxidation products, protein oxidation markers, and DNA damage marker (reviewed in Tóthová et al., 2015). Alternatively to measurements of oxidation products, a test measuring total oxidative status (TOS) was recently developed (Erel, 2005). Previous studies show that salivary TOS is increased in periodontitis patients and can be restored by periodontal therapy (Akalin et al., 2007; Wei et al., 2010). Oxidants produced during inflammatory response either react with target proteins or are neutralized by different antioxidants system. Therefore, measuring salivary total antioxidant capacity (TAC) can be also considered as an important tool for periodontal diagnostic. Some previous studies show that salivary TAC is decreased in periodontitis patients (Diab-Ladki et al., 2003; Mashayekhi et al., 2005; Guentsch et al., 2008), some studies report no significant difference in the salivary TAC between periodontitis patients and healthy controls (Brock et al., 2004; Tóthová et al., 2013), and one recent study shows an increase in salivary TAC in periodontitis patients (Almerich-Silla et al., 2015).

Oxidative stress in periodontitis arises from the immune response of the host to periodontal bacteria. Therefore, it would be interesting to investigate if there is some connection between salivary oxidative stress markers and the bacterial load. However, to date this relationship has not been investigated extensively (Almerich-Silla et al., 2015). Therefore, in the present study we investigated salivary TAC and TOS in periodontitis patients in relation to the levels of periodontal pathogens as well as in relation to clinical parameters of periodontitis.

## Materials and methods

### Study population and clinical periodontal examination

This cross-sectional study included 45 periodontitis patients and 37 periodontally healthy volunteers recruited from February 2010 until July 2012. The group of periodontitis patients included 15 patients with aggressive periodontitis and 30 patients with chronic periodontitis. All study participants gave written consent for participation in the study. The study protocol was approved by the ethics committee of the Medical University of Vienna (EK 623/2007).

Periodontal disease was diagnosed by experienced periodontologists with a postgraduate specialization in periodontology with clinical assessments using a periodontal probe and radiographs. Probing pocket depth (PD), clinical attachment loss (CAL), and bleeding on probing (BOP) were recorded at six sites per tooth using a periodontal probe (Hu-Friedy, Chicago, US). Bone loss was evaluated with intra-oral and panoramic radiographs. Periodontitis was classified according to the classification of the World Workshop 1999 (Armitage, 1999). Inclusion criteria for periodontitis patients were: ≥20 teeth, generalized aggressive periodontitis with a clinical attachment-loss ≥5 mm at minimum six different teeth, age of onset <35 years; and for those with generalized chronic periodontitis a clinical attachment-loss ≥5 mm at minimum six different teeth. In the control group, no radiographic bone loss and no probing pocket depth ≥4 mm was recorded. The control group exhibited no clinical signs of gingivitis. Exclusion criteria for all study participants were: systemic diseases, medication, periodontal or antibiotic treatment >6months prior to the investigation, pregnancy. Smoking status was recorded based on a questionnaire. Only current smoking status was considered, the amount of cigarette consumption was not recorded.

### Saliva collection

Unstimulated whole saliva samples were used in this study. Salivary collection was carried out between 8:00 and 11:00 a.m. Participants were asked to refrain from eating, drinking, smoking or brushing their teeth after midnight on the day of sampling. Unstimulated saliva was collected for 5 min according to the protocol of Navazesh et al. (2008), aliquoted and stored at −80°C until analysis.

### Total antioxidant capacity and total oxidant status measurements

TAC and total oxidant status (TOS) were measured by commercially available TAC kit and TOS kit, respectively (both Rel Assay Diagnostics, Turkey). Measurements were performed according to the manufacturer's instruction.

TAC assay was based on the measurements of the reduction of 2,2′-azino-bis (3-ethylbenzothiazoline-6-sulphonic acid; ABTS) radical. For the measurements of salivary TAC 225 μl of assay Reagent 1 (acetate buffer, pH 5.8) was mixed with 5 μl of saliva and the absorbance was measured at 420 nm after 30 s incubation. Afterwards, 20 μl of Reagent 2 (ABTS, 30 mM in acetate buffer, pH 3.6) were added into each sample and the absorbance at 420 nm was measured after 5 min incubation. TAC was calculated based on the differences in the absorbance at 420 nm before and after adding the Reagent 2. The assay was calibrated with trolox and the results were expressed in terms of mM trolox equivalent per liter (mmol trolox Equiv/L).

TOS of saliva samples were measured using a method developed by Erel (Erel, 2005). Briefly, 225 μL Reagent 1 (xylenol orange 150 μM, NaCl 140 mM and glycerol 1.35M in 25 mM H_2_SO_4_ solution, pH 1.75) was mixed with 35 μL of samples (saliva) and the absorbance of each sample was read spectrophotometrically at 560 nm as a sample blank. After that, 11 μL Reagent 2 (ferrous ion 5 mM and o-dianisidine10 mM in 25 mM H_2_SO_4_ solution) was added to the mixture, mixed for about 3–4 min and the last absorbance was read at 560 nm. TOS was calculated based on the differences in the absorbance at 560 nm before and after adding the Reagent 2. The assay was calibrated with H_2_O_2_ and the results were expressed in terms of μM H_2_O_2_ equivalent per liter (μmol H_2_O_2_ Equiv/L).

### Microbiological analysis

Salivary bacteria were measured using the method described previously (Haririan et al., 2014). *A. actinomycetemcomitans, P. gingivalis, T. forsythia, T. denticola*, and *F. nucleatum* in saliva were measured. Saliva in the tubes was centrifuged, and the supernatant was transferred into 2 mL tubes. DNA was extracted by means of a DNA Extraction Kit (ParoCheck®, Greiner Bio One, Kremsmuenster, Austria). Protein kinase K, buffer solution, and EtOH were added, and tubes were centrifuged, vortexed, and heated at 95°C. Part of the 16S rRNA gene was amplified by a highly conserved specific primer pair flanking the diversity box of each 16S rRNA gene. A chip (ParoCheckChip®, Greiner Bio One, Kremsmuenster, Austria) was used for hybridization, followed by washes at 50°C and drying by centrifugation. Finally, the DNA-Chip was analyzed by a scanner (CheckScanner) semi quantitatively using specific analytic software (Check Report Software Version 4.0.2, Greiner Bio One, Kremsmuenster, Austria). The signals describing the bacterial load in the samples were scored as −, (+), +, ++, or +++.

### Statistical analysis

Differences in TAC/TOS values between periodontitis and control groups were analyzed using Students *t*-test. The effect of diagnosis, age, gender, smoking status, and bacterial load on the salivary TAC and TOS was tested using multivariate generalized linear model. The differences in the detection frequency of particular bacteria, the proportion of female/male, and the proportion of smokers between groups were examined by χ^2^ test. Correlations between different parameters were checked by Spearmans's rank correlation tests. Differences were considered to be statistically significant at *p* < 0.05. Statistical power was calculated using freely available online tool (www.powerandsamplesize.com). All other statistical analysis was performed using statistical program SPSS 21.0.

## Results

### Demographic and clinical characterization of the study groups

Demographical characteristics and clinical parameters of healthy controls and periodontitis patients are listed in the Table 1. No significant difference in the proportion of female and male participants was found between the groups. The proportion of smokers was significantly higher in the periodontitis group compared to the control group (*p* < 0.01). Clinical parameters were significantly higher in the periodontitis group than in the control group (*p* < 0.01). The results of the semiquantitative analysis of *A. actinomycetemcomitans, P. gingivalis, T. denticola, T. forsythia, and F. nucleatum* in saliva of healthy individuals and periodontitis patients are shown in the Figure 1. Significantly higher detection rates of *P. gingivalis, T. denticola*, and *T. forsythia*, were observed in periodontitis patients compared to healthy subjects (*p* < 0.01). No significant difference in the detection rate of *A. actinomycetemcomitans* (*p* = 0.051) and *F. nucleatum* (*p* = 0.642) was found between periodontitis and control groups.

**Table 1:** **Demographic characteristics and clinical parameters of the study population**.

	**Control**	**Periodontitis overall**	**Aggressive periodontitis**	**Chronic periodontitis**
Number of participants	37	45	15	30
Age, years	36.1 ± 10.3	45.2 ± 9.8	34.7 ± 5.8	50.5 ± 6.6
Female/male (n)	21/16	17/28	5/10	12/18
Non-Smoker/Smoker (n)	25/12	15/30^#^	6/9	9/21
PPD, mm	1.71 ± 0.21^*^	3.88 ± 0.86	3.91 ± 1.09^*^	3.86 ± 0.75^*^
CAL, mm	1.74 ± 0.22^*^	4.43 ± 1.12	4.59 ± 1.17^*^	4.41 ± 0.97^*^
BOP, %	2.6 ± 3.1^*^	41.0 ± 25.1	45.7 ± 27.3^*^	38.7 ± 24.0^*^
Number of teeth with PPD ≥ 5 mm	0	19.2 ± 6.2^*^	20.3 ± 6.9^*^	18.6 ± 5.8^*^

*Data are presented as mean ± SD. BOP, bleeding on probing; PPD, probing pocket depth; CAL, clinical attachment loss*.

* *Significantly different vs. control group, p < 0.05, t-test*.

# *significantly different vs. control group, p < 0.05, χ^2^ test*.

**Figure 1:**
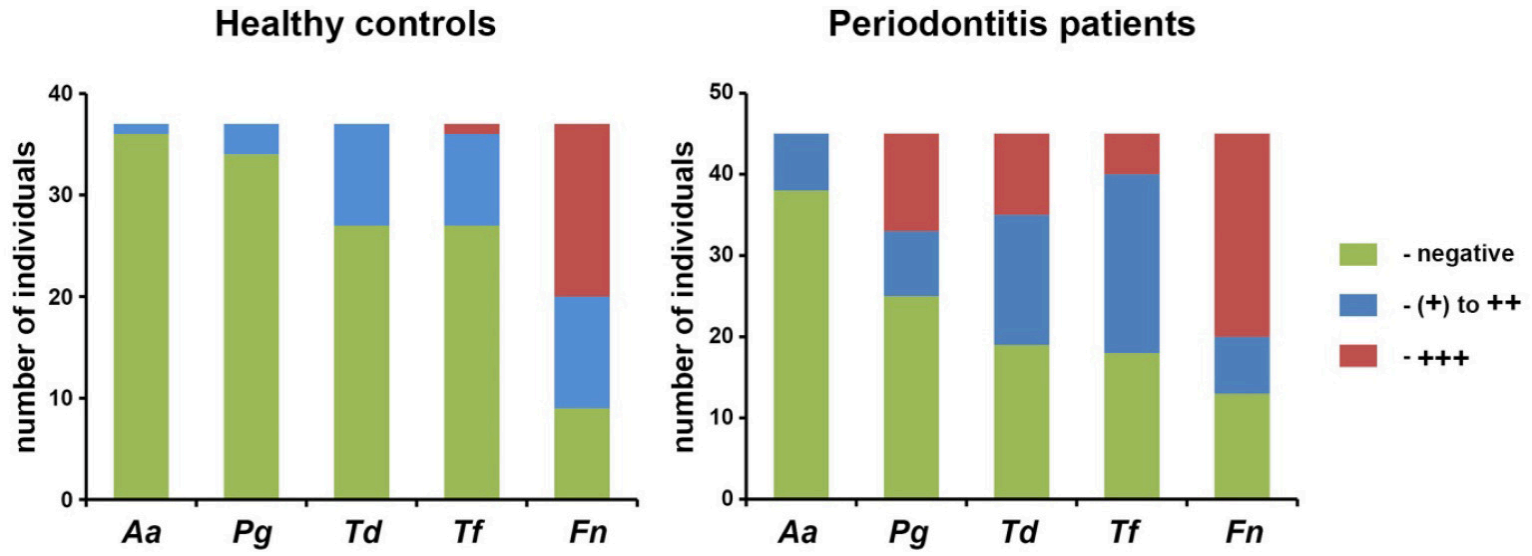
**Detection of different periodontal pathogens in saliva of healthy individuals and periodontitis patients**. The results of semiquantitative analysis of the presence of periodontal pathogens in the saliva are presented. The detection rate of *P. gingivalis (Pg), T. denticola (Td)*, and *T. forsythia (Tf)* was significantly higehr in periodontits patients than in control group. No significant difference in the detection rate of *A. actinomycetemcomitans* and *F. nucleatum* was detected.

### Salivary TAC and TOS

**Figure 2:**
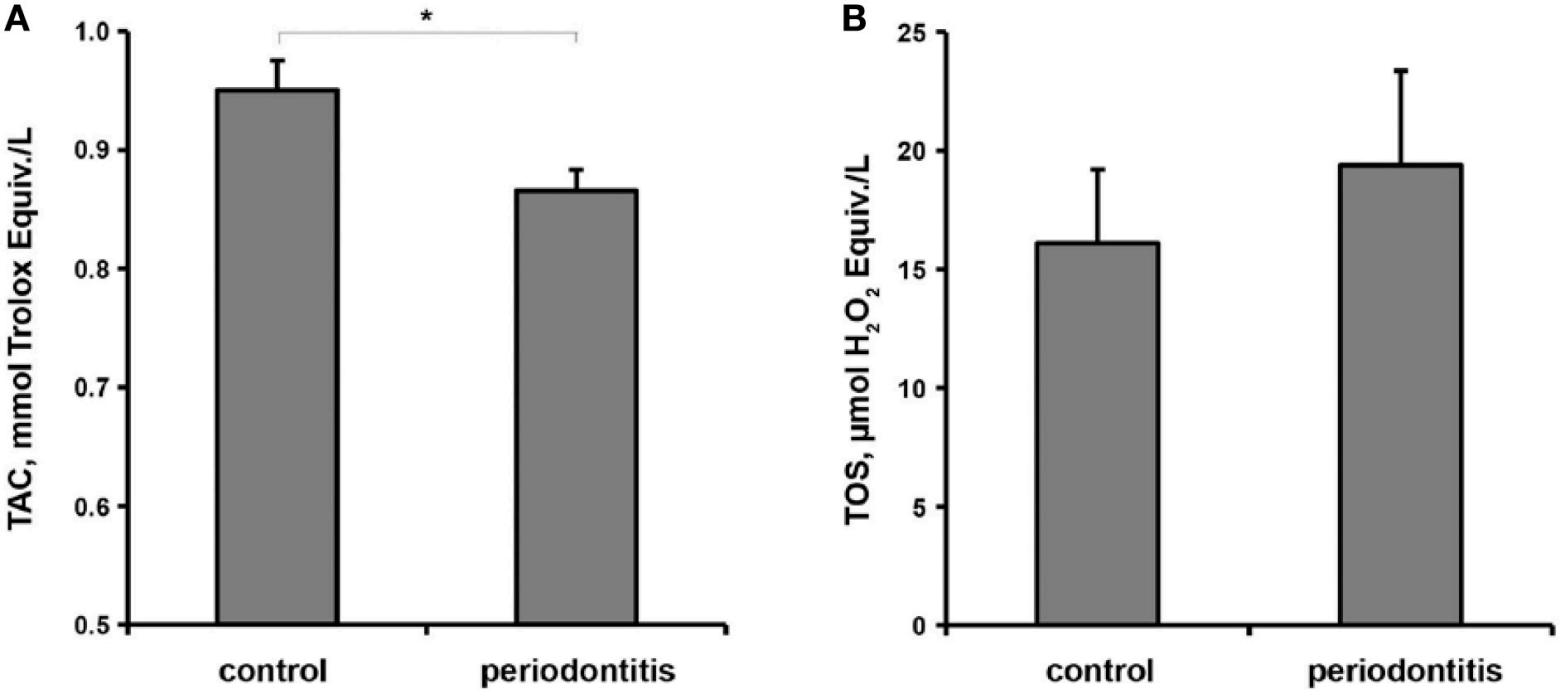
**Oxidative stress parameter in the control and periodontitis groups**. Salivary total antioxidant capacity **(A)** and total oxidant status **(B)** are presented as mean ± s.e.m. ^*^Significantly different vs. control group, *p* < 0.05, *t*-test.

The results of a multivariate generalized linear model analysis of the effect of overall diagnosis, smoking status, gender, age, and bacterial load on the salivary TAC and TOS are presented in the Table 2. Multivariate test showed a significant dependency of study parameters on the overall diagnosis. Between subject tests showed that only overall diagnosis has a significant effect on the salivary TAC, whereas age, gender, smoking status, and bacterial load had no significant effect on this parameter. None of the factors had a significant effect on salivary TOS. The salivary TAC and TOS in control and periodontitis groups are shown in the Figure 2. The salivary TAC values in periodontitis group were significantly lower compared to the control group (*p* < 0.05, power = 0.82). No significant difference in the salivary TOS between control and periodontitis groups was found. No significant difference in the salivary TAC and TOS was found between patients with aggressive and chronic periodontitis (data not shown).

**Table 2:** **Effect of overall diagnosis, gender, smoking status, age, and bacterial load on the salivary TAC and TOS analyzed by multivariate generalized linear model**.

	**Multivariate test Wilks-Lambda**		**Salivary TAC**			**Salivary TOS**		
	***F*-value**	***P*-value**	***F*-value**	***P*-value**	**β**	***F*-value**	***P*-value**	**β**
Overall diagnosis “0”–control “1”–periodontitis	5.104	0.014	5.289	0.028	−0.386	0.138	0.713	0.139
Gender “0”–male “1”–female	1.042	0.368	2.990	0.093	0.160	0.037	0.848	−0.088
Smoking “0”–non-smokers “1”–smokers	1.498	0.244	2.272	0.141	0.045	0.141	0.731	0.124
Age	1.381	0.118	1.611	0.085	0.021	1.047	0.449	0.013
*A. actinomycetemcomitans* “0”–negative; “1”− ≤ ++ “2”−+++	0.554	0.765	0.001	0.981	0.063	0.161	0.691	−0.107
*P. gingivalis* “0”–negative; “1”− ≤ ++ “2”−+++	1.543	0.184	0.120	0.888	−0.204	0.491	0.616	0.263
*T. denticola* “0”–negative; “1”− ≤ ++ “2”−+++	1.322	0.256	3.384	0.064	0.355	2.707	0.082	−0.401
*T. forsythia* “0”–negative; “1”− ≤ ++ “2”−+++	1.418	0.214	0.639	0.639	−0.098	0.952	0.396	−0.107
*F. nucleatum* “0”–negative; “1”− ≤ ++ “2”−+++	1.113	0.369	1.529	0.232	−0.176	0.250	0.780	0.282

*Parameters are calculated by multivariate generalized linear models with TAC and TOS as dependent variable and overall diagnosis, gender, smoking status, age, or bacterial load as independent variables. β, standardized regression coefficient*.

### Correlation of TAC and TOS with clinical parameters of periodontitis

**Figure 3:**
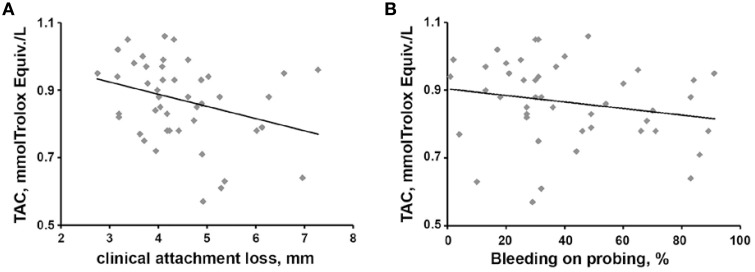
**Relationship between salivary TAC and clinical parameters in periodontitis patients**. Relationships salivary TAC vs. CAL **(A)** and salivary TAC vs. BOP **(B)** are presented. Each point represents one periodontitis patient. Spearman rank correlation test revealed a significant negative correlation between salivary TAC and CAL (*r* = −0.312, *p* < 0.05) and a non-significant negative correlation between salivary TAC and BOP (*r* = −0.258, *p* = 0.087).

In periodontitis patients, the salivary TAC exhibited a significantly negative correlation with CAL parameter (*r* = −0.312, *p* < 0.05, see Figure 3A). A negative but non-significant correlation was observed between salivary TAC and BOP (*r* = −0.258, *p* = 0.087, see Figure 3B). No significant correlation of TAC with clinical parameters BOP, PPD, and the number of teeth with PPD ≥ 5 mm was found. No significant correlation of salivary TOS with any clinical parameter was found.

### Clinical parameters and salivary TAC/TOS in relation to bacterial load

**Figure 4:**
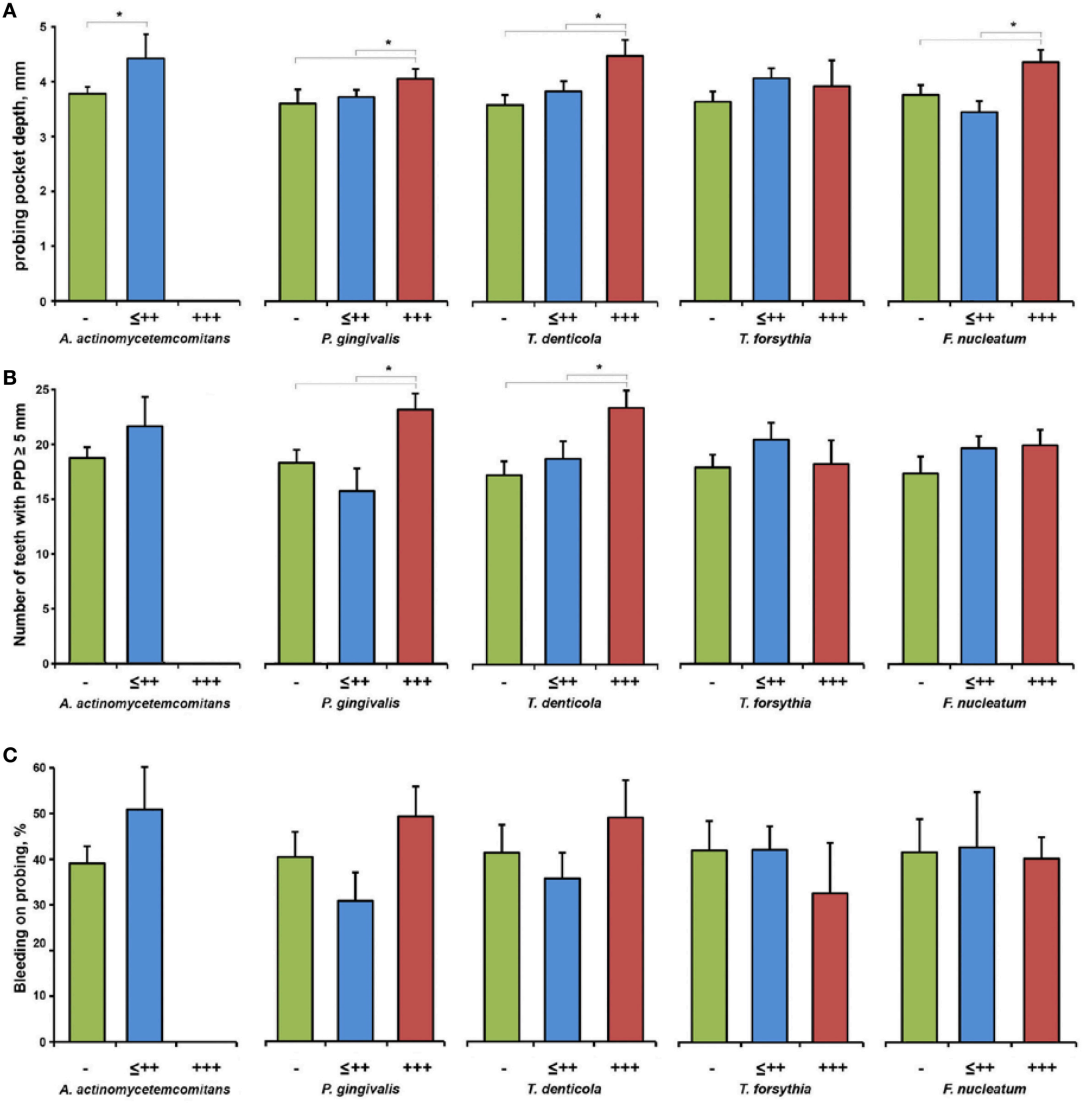
**Clinical parameters of periodontitis patients depending on the bacterial load**. PPD **(A)**, number of teeth with PPD ≥ 5 mm **(B)**, and BOP **(C)** in subgroups of periodontitis patients based on the results of the semiquantitative bacterial analysis in the saliva: negative (group “−”), from (+) to ++ (group “≤ ++”), and +++ (group “+++”). Data are presented as mean ± s.e.m. ^*^Significantly different with *p* < 0.05.

The dependency of PPD, number of teeth with PPD ≥ 5 mm, and BOP on the bacterial load measured in the saliva of periodontitis patients is shown in Figure 4. Increased salivary content of *A. actinomycetemcomitans, P. gingivalis, and T. denticola* was associated with significantly higher PPD and the number of teeth with PPD ≥ 5 mm in periodontitis patients. No significant effect of the salivary presence of bacteria on BOP (Figure 4C) and CAL (data not shown) was observed.

**Figure 5:**
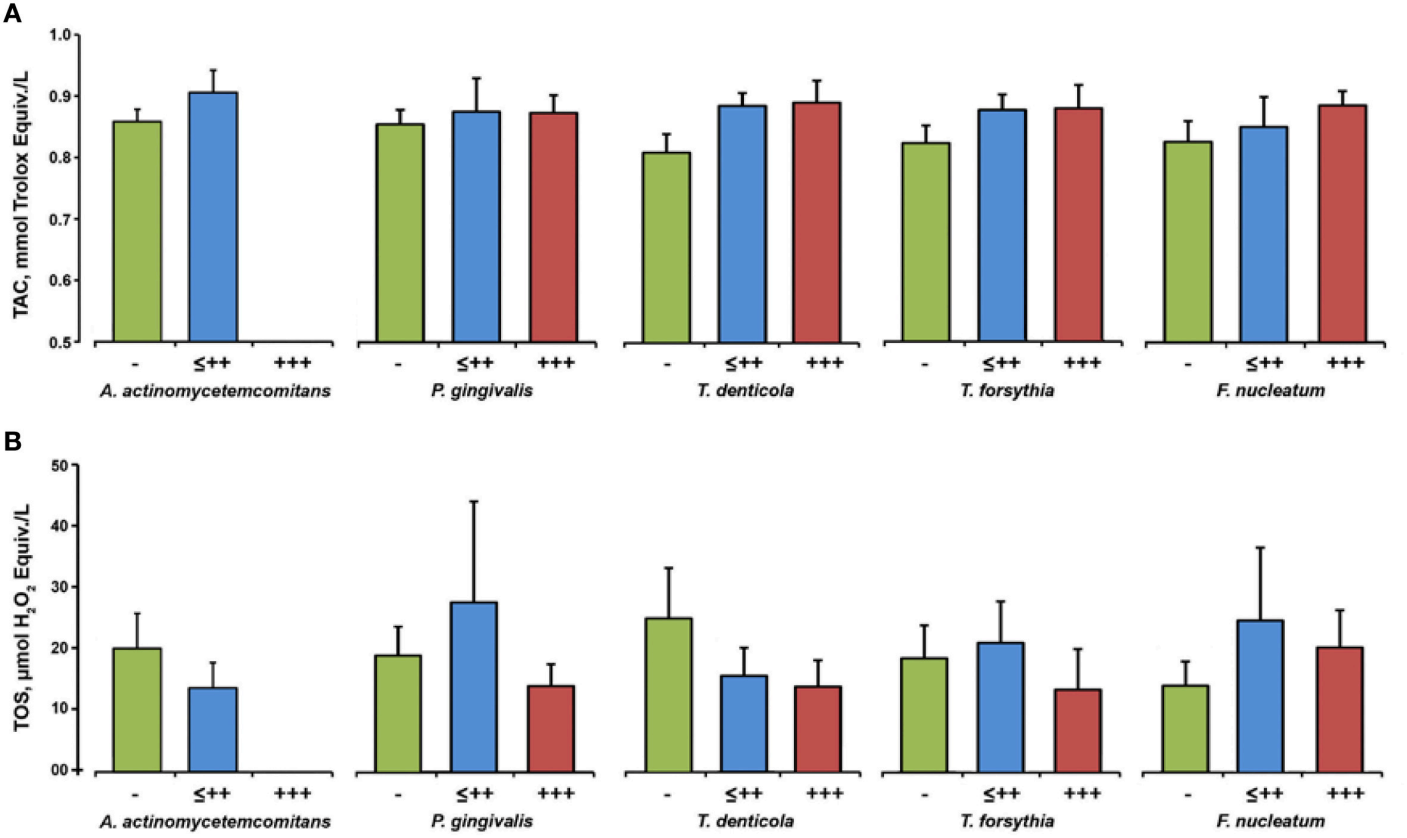
**Oxidative stress parameters depending on the bacterial load**. Salivary TAC **(A)** and TOS **(B)** in subgroups of periodontitis patients based on the results of the semiquantitative bacterial analysis in the saliva: negative (group “−”), from (+) to ++ (group “≤ ++”), and +++ (group “+++”). Data are presented as mean ± s.e.m.

Figure 5 shows salivary TAC and TOS depending on the presence of different bacteria in the saliva of periodontitis patients. No significant effect of any periodontal bacteria on the salivary TAC (Figure 5A) and salivary TOS (Figure 5B) was observed.

## Discussion

In the present study we investigated the differences in TAC and TOS in saliva in individuals with periodontitis and healthy controls and investigated their relationship with clinical parameters of periodontitis and the presence of periodontopathic bacteria *A. actinomycetemcomitans, P. gingivalis, T. forsythia, T. denticola*, and *F. nucleatum* in saliva. Activation of the host response by periodontal pathogens results in an activation and infiltration of neutrophils, which are the primary source of ROS in periodontitis (Ryder, 2010; Scott and Krauss, 2012). ROS production by neutrophils is considered as an important mechanism of bacteria killing (Nauseef, 2007). However, ROS production by neutrophils might also cause damages of host tissues when ROS are not neutralized by the antioxidant system or in case of an impaired neutrophils clearance (Hajishengallis and Hajishengallis, 2014). A proper balance between ROS production and TAC of the host tissue plays an important role in the homeostasis of periodontal tissue and prevents tissue destruction upon activation of the immune system by periodontal pathogens.

Analysis using multivariate generalized linear model showed that salivary TAC was significantly dependent on the overall diagnosis but not on other parameters such as age, gender, smoking status, and bacterial load. None of the parameters, such as overall diagnosis, age, gender, smoking status, and bacterial load exhibited a significant effect on salivary TOS. The antioxidant capacity of saliva is due to low molecular antioxidants such as uric acid, ascorbic acid, and albumin (Moore et al., 1994) as well as due to antioxidant enzymes such as superoxide dismutase, catalase, and glutathione peroxidase (Battino et al., 2002). Salivary TOS might be accounted by different reactive oxygen and nitrogen species, such as hydroxyl radical, hydrogen peroxide, or peroxynitrite (Takahama et al., 2005; Chitra et al., 2012). Our data suggest that changes in the oxidative stress parameters in periodontitis patients are presumably associated with the inflammatory processes in periodontitis and not directly with a bacterial load. The contribution of other factors such as age, gender, and smoking status on the oxidative stress parameters must be considered in the future studies with higher number of patients.

Salivary TAC in periodontitis patients was significantly lower than those in healthy subjects. There was a significant negative relationship between salivary TAC and CAL. Moreover, a tendency of a negative relationship of salivary TAC with BOP was also observed. Our results are supported by some previous studies showing that salivary TAC is lower in periodontitis patients and negatively correlates with periodontitis severity (Diab-Ladki et al., 2003; Mashayekhi et al., 2005; Guentsch et al., 2008). Immune response against periodontal pathogens is associated with an enhanced production of ROS by neutrophils and macrophages (for review, see Chapple and Matthews, 2007). To avoid host tissue destruction these ROS are neutralized by antioxidants, which might result in decreased TAC. Systemic and local TAC in periodontitis patients might reflect increased oxygen radical activity during periodontal inflammation and can be restored to control subject levels by non-surgical therapy (Chapple and Matthews, 2007). However, there are also some studies showing that salivary TAC is either increased or remains on the same level in periodontitis patients compared to healthy controls (Brock et al., 2004; Tóthová et al., 2013; Almerich-Silla et al., 2015). The differences in the results of TAC between different studies could be explained by different analytical methods used by TAC assessment (reviewed in Wang et al., 2015). Particularly, in our study, TAC was measured based on the ability of saliva sample to reduce ABTS radical, whereas in another study TAC measurements were based on the saliva ability to prevent ABTS oxidation (Almerich-Silla et al., 2015). It should be also noted that TAC is a complex parameter which includes the integrated activity of different antioxidants and often depends on their interaction and synergistic effects (Ghiselli et al., 2000).

We did not find any significant difference in the salivary TOS between periodontitis patients and healthy controls. Salivary TOS exhibited no correlation with clinical parameters and the bacterial load. This observation is in contrast to recent studies reporting salivary TOS in periodontitis patients of about 1.5 times higher compared to healthy individuals as well as a strong correlation of TOS with clinical parameter of periodontitis (Akalin et al., 2007; Wei et al., 2010). However, in these two studies, patients' selection was more restrictive: they include only never smokers, all studies participants have similar age, socio-economic status and nutrition habits. Therefore, patient selection could be a crucial parameter influencing salivary levels of TOS and this fact is rather limiting the use of salivary TOS as a marker of periodontal disease.

An assumption that an increased salivary TOS reflects an increased production of ROS in periodontitis is rather questionable. Most ROS produced by neutrophils and macrophages either reacts with target proteins or are neutralized by antioxidants. Furthermore, the production of ROS as a result of an inflammatory reaction occurs either before or at the same time of tissue damages (Halliwell, 2000). Therefore, lipid peroxidation products malondialdehyde (MDA) and thiobarbituric acid-reacting substances (TBARS), advanced oxidation protein products (AOPP), and oxidative DNA damage product 8-hydroxydeoxyguanosine (8-OHdG) are often used as an indicator of oxidative stress (Chapple and Matthews, 2007; Tóthová et al., 2015; Wang et al., 2015). Previous studies suggest that salivary TBARS and 8-OHdG levels are enhanced in periodontitis patients compared to controls (for review, see Tóthová et al., 2015). Two previous studies show a significant positive correlation between the levels of periodontal pathogens and oxidative stress markers in saliva from periodontitis patients (Sawamoto et al., 2005; Almerich-Silla et al., 2015). Moreover, analysis with predictive model shows that salivary markers of lipid peroxidation and DNA damage might have good diagnostic potential for periodontitis (Almerich-Silla et al., 2015).

We have determined the levels of different bacteria in saliva. As shown by our previous study, salivary bacteria levels exhibit a good correlation with the subgingival bacterial load in the dental plaque of periodontitis patients (Haririan et al., 2014). We observed that the salivary levels of *P. gingivalis, T. forsythia*, and *T. denticola* were significantly enhanced in periodontitis patients compared to healthy subjects. This confirms the previous finding suggesting the importance of red complex bacteria as true pathogens in adult periodontitis patients (Socransky et al., 1998). We further found that a high bacterial load was associated with increased clinical parameters of periodontitis such as PPD and BOP but exhibited no correlation with salivary TAC and TOS. However, a negative correlation of salivary TAC with CAL and BOP was observed. Our observations suggest that changes in salivary antioxidant capacity are associated rather with the inflammatory response than with an enhanced bacterial load. This conclusion is generally in line with the current opinion that periodontal tissue destruction is mainly due to a dysregulated unresolved immune response than due to an enhanced bacterial load (Hasturk and Kantarci, 2015; Meyle and Chapple, 2015).

As well-known, periodontitis is a local inflammatory disease with multifactorial character, with fluctuations in bacterial burden, systemic host immune response, and tissue destruction. Up to date, various host-derived factors in periodontitis can be detected in saliva, which provide important information regarding the status of periodontal tissues. Most of the studies focused on biomarkers, which hold a potential diagnostic significance relevant to three important biological phases of periodontal disease, i.e., well-known markers C-reactive protein, IL-1β, IL-6, TNF-α involved in inflammatory phase; matrix metalloproteinases (MMP-8 and MMP-9) in connective-tissue degradation phase and alkaline phosphatase, receptor activator of NF-κB ligand, and osteoprotegerin in bone-turnover phase (for review, see Miller et al., 2010). Moreover, our previous studies demonstrate other biomolecules such as histamine, chromogranin A (CgA), melatonin, and nitric oxide derivatives to be related to the periodontal bacterial load and severity of periodontitis, as well as some of them were restored by a non-surgical therapy and influenced by smoking and gender (Bertl et al., 2012, 2013; Haririan et al., 2012; Andrukhov et al., 2013).

Before considering oxidative stress parameters as potential markers of periodontitis, it is important to take into account their dependency on different factors such as age, smoking, gender, and nutrition. We did not find any effect of gender on both TAC and TOS. Gender is a well-known risk factor of periodontitis and other oxidative stress related disease (Genco, 1996). Male individuals usually have a higher prevalence and severity of periodontal disease than females (Shiau and Reynolds, 2010). In our previous study we also indicated male gender should be considered an important factor by assessing the risk of CVDs in periodontitis patients (Andrukhov et al., 2013). A recent study reports that changes of TAC and oxidative stress marker TBARS in saliva of periodontitis patients might be gender dependent (Baňasová et al., 2015). Antioxidant protection in females is usually higher due to an increased activity of antioxidant enzymes in pre-menopausal females when compared to men (Pepe et al., 2009; Bloomer and Fisher-Wellman, 2010). Various markers of oxidative stress such as MDA, AOPP, nitric oxide derivatives, as well as Trolox-Equivalent Antioxidant Capacity (TEAC) are reported to differ between men and women (Andrukhov et al., 2013; Bloomer and Lee, 2013). However, the exact effect of gender on salivary TAC and TOS must be investigated in further studies with higher patients' numbers.

Smoking is another important factor influencing the salivary levels of oxidative stress parameters. Smoking stimulates the oxidative burst of neutrophils and therefore influences the oxidative balance in the whole body (Ryder et al., 1998). In our study we did not observe any significant effect of smoking on salivary TAC and TOS. Similarly to our observation, some previous studies also do not observe any significant effect of smoking on salivary TAC (Charalabopoulos et al., 2005; Guentsch et al., 2008). In contrast, another study reports that the activity of antioxidant enzymes superoxide dismutase, glutathione peroxidase, and catalase is increased in smoking periodontitis patients compared to non-smokers (Tonguç et al., 2011). Salivary levels of lipid peroxidation marker MDA are reported to be higher in smoking patients than in non-smokers (Guentsch et al., 2008; Tonguç et al., 2011). The dependency of oxidative stress markers on current smoking status, smoking history, and smoking intensity (cigarette per day or pack per years) must be taken into account before considering these parameters as candidate for periodontal disease markers.

Age might also influence the salivary levels of oxidative stress parameters. Previous studies suggest that the levels of salivary antioxidants as well as protein oxidation products positively correlate with the age (Hershkovich et al., 2007; Celecová et al., 2013). However, these changes seem to be pronounced mainly in the elderly individuals. In our study, we did not observe any significant effect of age on the salivary TAC and TOS. Periodontitis patients included in our study were about 9 years older than healthy controls. The observed differences in salivary TAC observed in our study cannot be contributed to the different age on the salivary because older individual have higher TAC (Hershkovich et al., 2007; Celecová et al., 2013) but we observed the lower TAC in periodontitis patients.

In conclusion, the present study showed that salivary TAC is decreased in periodontitis patients compared to healthy controls. The change of TAC correlates with the severity of periodontal disease but not with the bacterial load, which implies that changes in the oxidative status in periodontitis patients are rather due to dysregulated immune response than an increased bacterial load.

## Author contributions

Designed research: TZ, OA, HH, XR. Performed research and analyzed data: TZ, OA, HH, MM, SL. Edited the manuscript: HH, MM, SL, ZL. Wrote the manuscript: TZ, OA, XR.

### Conflict of interest statement

The authors declare that the research was conducted in the absence of any commercial or financial relationships that could be construed as a potential conflict of interest.
